# A history of obesity leaves an inflammatory fingerprint in liver and adipose tissue

**DOI:** 10.1038/ijo.2017.224

**Published:** 2017-10-24

**Authors:** I P Fischer, M Irmler, C W Meyer, S J Sachs, F Neff, M Hrabě de Angelis, J Beckers, M H Tschöp, S M Hofmann, S Ussar

**Affiliations:** 1 JRG Adipocytes and Metabolism, Institute for Diabetes and Obesity, Helmholtz Diabetes Center at Helmholtz Center Munich, Garching, Germany; 2 German Center for Diabetes Research (DZD), München-Neuherberg, Germany; 3 Division of Metabolic Diseases, Department of Medicine, Technische Universität München, Munich, Germany; 4 Institute for Experimental Genetics, Helmholtz Zentrum München, München-Neuherberg, Germany; 5 Medizinische Klinik und Poliklinik IV der LMU, Munich, Germany; 6 Institute for Diabetes and Regeneration, Helmholtz Diabetes Center at Helmholtz Zentrum München, Garching, Germany; 7 Institute for Pathology, Helmholtz Zentrum München, München-Neuherberg, Germany; 8 Technische Universität München, Lehrstuhl für Experimentelle Genetik, Freising, Germany; 9 Institute for Diabetes and Obesity, Helmholtz Diabetes Center at Helmholtz Zentrum München, Garching, Germany

## Abstract

**Background/Objectives:**

Dieting is a popular yet often ineffective way to lower body weight, as the majority of people regain most of their pre-dieting weights in a relatively short time. The underlying molecular mechanisms driving weight regain and the increased risk for metabolic disease are still incompletely understood. Here we investigate the molecular alterations inherited from a history of obesity.

**Methods:**

In our model, male high-fat diet (HFD)-fed obese C57BL/6J mice were switched to a low caloric chow diet, resulting in a decline of body weight to that of lean mice. We measured body composition, as well as metrics of glucose, insulin and lipid homeostasis. This was accompanied by histological and gene expression analysis of adipose tissue and liver to assess adipose tissue inflammation and hepatosteatosis. Moreover, acute hypothalamic response to (re-) exposure to HFD was assessed by qPCR.

**Results & Conclusions:**

Within 7 weeks after diet switch, most obesity-associated phenotypes, such as body mass, glucose intolerance and blood metabolite levels were reversed. However, hepatic inflammation, hepatic steatosis as well as hypertrophy and inflammation of perigonadal, but not subcutaneous, adipocytes persisted in formerly obese mice. Transcriptional profiling of liver and perigonadal fat revealed an upregulation of pathways associated with immune function and cellularity. Thus, we show that weight reduction leaves signs of inflammation in liver and perigonadal fat, indicating that persisting proinflammatory signals in liver and adipose tissue could contribute to an increased risk of formerly obese subjects to develop the metabolic syndrome upon recurring weight gain.

## Introduction

Excessive accumulation of fat, specifically in the visceral fat depots, deregulates systemic lipid- and glucose-homeostasis,^1^ contributing to the development of type 2 diabetes, non-alcoholic fatty liver disease (NAFLD), cardiovascular and neoplastic diseases.^1^ In addition to food intake and exercise, genetics in combination with a diverse set of environmental factors can result in epigenetic modifications and changes in gut microbiota, and thereby contribute to the development of obesity and the risk to develop the metabolic syndrome.^2, 3^ Albeit the molecular details and the temporal order of events are still under investigation, an inability of adipose tissue to store excessive calories as triglycerides results in spillover of lipids into other organs, foremost the liver, as well as local and systemic inflammation. This sets off a cascade of metabolic alterations, resulting in systemic insulin resistance, dyslipidemia and the development of the metabolic syndrome. White adipose tissue is comprised of anatomically distinct depots, with different cardiometabolic risk associations.^4^ In general accumulation of subcutaneous adipose tissues harbors little to no risk towards the development of metabolic complications, whereas increased visceral adipose tissue predisposes to the development of the metabolic syndrome.^4^ The reasons for these different risk associations result from differences in the way excessive calories are stored, hyperplasia vs hypertrophy,^5^ developmental origin,^6^ glucose and lipid metabolism,^7^ and vascularization.^8^ All these factors contribute to an increased inflammatory state and increased lipid spillover from visceral adipose depots into other organs. Moreover, differences in adipokine secretion from different depots directly impact on food intake, glucose homeostasis and other metabolic functions.^9^ Importantly, obesity-associated hepatosteatosis is the first step towards hepatic insulin resistance and NAFLD, posing significant health risks.^10^

Thus, multiple invasive and non-invasive therapeutic strategies exist to treat obesity.^1, 11, 12^ They all have in common that already moderate weight loss of ~10% significantly improves metabolic health.^13^ Bariatric surgery is the most efficient approach to reduce body mass, with a long-term reduction of >50% of pre-operative body mass.^11, 14^ Concurrently co-morbidities such as type 2 diabetes, hypertension and dyslipidemia are mitigated by bariatric surgery.^15^ However, surgical-based weight loss bares the risk of post-operative adverse effects, such as dumping syndrome, consequent malabsorption and impaired nutritional status, bowel injuries and ulcera,^16, 17^ limiting the access to these procedures for many patients.

Conversely, dieting is a much more popular, yet less effective way to lose weight, especially in overweight to moderately obese subjects.^12, 18^ Unfortunately, the vast majority of people regain most of their lost weight within 1–5 years post dieting and in many cases exceed their pre-diet body mass.^19, 20, 21^ Multiple components determine post dieting body weight regain, such as a reduction in resting energy expenditure^22, 23^ and increased hunger,^24^ through reprogramming of orexigenic pathways.^25, 26, 27^ The metabolic consequences of body weight regain are much less understood and several clinical studies have revealed somewhat contradicting results. However, multiple studies showed that already a regain of >2% of the pre-dieting weight reverses most of the metabolically beneficial effects of weight loss. This indicates that some sort of memory of past obesity remains, predisposing the body to metabolic alterations upon weight regain.^28^ Indeed, Zamarron *et al.*^29^ recently reported that voluntary weight loss in obese C57Bl/6 mice resulted in persistent adipose tissue fibrosis and increased macrophage infiltration in perigonadal adipose tissue up to six months after diet intervention, further suggesting a tissue intrinsic memory of past obesity.

In this context, we describe the specific molecular alterations inherited from a history of obesity, to characterize underlying predispositions of formerly obese subjects to the development of the metabolic syndrome upon recurrent weight gain. We show that a history of obesity does not facilitate hyperphagia within 48 h of high-fat diet (HFD)- refeeding, but leaves an inflammatory imprint in liver and perigonadal fat, despite normalization of most metabolic parameters.

## Materials and methods

### Animals

Six-week-old male C57BL/6J mice (Charles River) were imported in our animal facility at constant ambient temperature and humidity with a 12 h light–dark cycle. After 2 weeks of acclimatization, mice were randomly assigned to HFD (obese, *n*=47) or the control diet (lean, *n*=23) groups. Mice of the HFD group received a high-fat high-sucrose diet (Research Diets D12331, 58% kcal from fat) for 20 weeks, while mice of the lean group were given the low-fat control diet (LFD; Research Diets D12329, 10.5% kcal from fat). After 20 weeks of feeding, a group of HFD-fed mice was switched to LFD (formerly obese, *n*=23). A cohort of 35-week-old formerly obese and lean mice were (re-) introduced to HFD *ad libitum* for 48 h (formerly obese-HFD and lean-HFD, *n*=12). Sample sizes were calculated based on expected effect sizes and variance. All animal experiments were approved by the German Animal Welfare Authorities.

### Metabolic measurements

Plasma and liver triglycerides, cholesterol and non-esterified free fatty acids were determined via the LabAssay kits (WAKO Chemicals, Osaka, Japan). Plasma insulin and leptin were measured using the Ultrasensitive insulin and leptin ELISA kits (ALPCO Diagnostics, Salem, MA, USA), adiponectin was measured via ELISA (Merck Millipore, Billerica, MA, USA).

### NMR-analysis

Body composition was assessed by nuclear magnetic resonance measurements (EchoMRI LLC, Houston, TX, USA).

### Glucose tolerance test

Mice were fasted for 6 h during the light phase and basal glucose levels (0 min) were determined, using a FreeStyle Freedom Lite Glucometer (Abbot, Wiesbaden, Germany). Thereafter, 2 g per kg body weight glucose was injected intraperitoneally and blood glucose was assessed at 15, 30, 60 and 120 min.

### Cytochrome C oxidase (COX) activity

BAT (30 mg) was homogenized in tissue buffer with a Potter-type homogenizer and sonicated with several short bursts, as previously described.^30^ COX activity was determined from 120 μg homogenized BAT at 37 °C by recording the oxygen consumption with an Oxygraph O2K (Oroboros Instruments, Innsbruck, Austria) as described.^30^ O_2_ consumption values are given as pmol/(s*mg BAT tissue).

### Histology

Tissues were fixed in 4% paraformaldehyde (Carl Roth, Karlsruhe, Germany) at room temperature for 24 h, dehydrated and embedded in paraffin (Leica, Wetzlar, Germany). Sections at 4 μm were prepared using a microtome (Leica). Adipocyte size distribution was determined from H&E-stained sections of scWAT and gWAT. Pictures from three different areas per tissue were taken (AxioCam MRC, × 200 magnifications). Adipocyte cell size was determined by measuring 20–25 randomly picked adipocytes per area of the respective adipose tissue using AxioVision Rel.4.8. Number of crown-like structures were counted and normalized per total number of counted adipocytes (*n*=4–5).

Masson’s trichrome staining was performed on paraffin-embedded tissue sections (*n*=5–8), using the Trichrome stain (Masson)-Kit (HT15) (Sigma-Aldrich, Darmstadt, Germany).

Fibrosis (0=no fibrosis, 6=cirrhosis) and steatosis grading (1=<5% of liver cells involved, 4=>66% liver cells involved) were performed according to standard guidelines.^31, 32^ The histological score was calculated by summing up the assessed fibrosis score and steatosis score. All histological assessments were performed double-blinded.

### RNA analysis

RNA was extracted using an RNeasy Kit (Qiagen, Venlo, Netherlands), according to the manufacturer’s instruction. RNA (1 μg) was reverse transcribed to cDNA using the QuantiTect Reverse Transcription Kit (Qiagen) according to the manufacturer’s protocol. qPCR was performed using SYBR green (Life Technologies, Darmstadt, Germany) on a ViiA 7 Real-Time PCR System (Applied Biosystems, Thermo Fisher Scientific, Waltham, MA, USA). Primer sequences are enlisted in Supplementary Table 14. Differential expression is calculated as ΔΔct.^33^ RNA integrity was determined using the Agilent RNA6000 Pico kit in the Agilent 2100 Bioanalyzer, according to the manufacturer’s instructions. RNA with a RIN >7 was used for microarray analysis. Total RNA (30 ng) was amplified (Ovation PicoSL WTA System V2) in combination with the Encore Biotin Module (Nugen, San Carlos, CA, USA). Amplified cDNA was hybridized on Affymetrix Mouse Gene 2.0 ST arrays. Staining and scanning (GeneChip Scanner 3000 7G) was done according to the Affymetrix expression protocol including minor modifications as suggested in the Encore Biotin protocol.

### Statistical analysis

Data are presented as mean±standard error of the means (s.e.m.), unless stated differently in the figure legend. Data were tested for normality via D’Agostino & Pearson omnibus, Shapiro–Wilk and KS normality test. For Gaussian distributed data, statistical significance was determined by unpaired two-sided Student’s *t*-test or, for multiple comparisons, using one- or two-way ANOVA, followed by Tukey’s test. For nonparametric data, significance was determined via Kruskall–Wallis test followed by Dunn’s Multiple Comparison’s Test. Liver grading was tested by X^2^-test. Differences reached statistical significance with *P*<0.05. For the gene array analysis, the Expression Console (v.1.4.1.46, Affymetrix) was used for quality control and to obtain annotated normalized RNA gene-level data (standard settings including median polish and sketch-quantile normalization). Genewise testing for differential expression was done employing the limma *t*-test and Benjamini–Hochberg multiple testing correction (false discovery rate (FDR) <10%). Sets of regulated genes were defined by *P*<0.01 (limma *t*-test) or FDR<10% (Benjamini–Hochberg) and further filtered for FC (>1.2 × for liver and >1.3 × for gWAT) and average expression in at least one group of the data set (>8 for liver and >16 for gWAT). Heatmaps were generated with the R script pheatmap. In case of several probe sets for the same gene, only the one with the highest ratio is shown. The pathway analyses were generated through the use of QIAGEN’s Ingenuity Pathway Analysis (Qiagen). The Fisher’s Exact Test was used to define sets of enriched canonical pathways (*P*<0.05) or biological functions (*P*<0.01). Statistical analyses were performed by utilizing the statistical programming environment R,^34^ implemented in CARMAweb (CARMAweb version 1.5.18—uses R version 2.11.0 together with Bioconductor version 2.6),^35^ GraphPad Prism 6 and SPSS Version 22.

## Results

### Weight loss reverses diet-induced obesity phenotype

**Figure 1:**
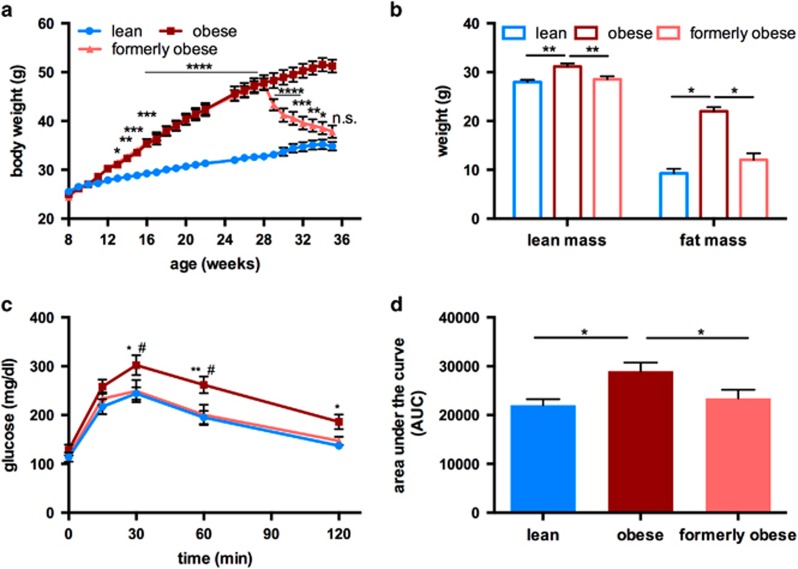
*Ad libitum* switch to low-fat diet reverses diet-induced obesity phenotype of male C57BL/6J mice. (**a**) Body weight curve of lean (*n*=23), obese (*n*=24) and formerly obese mice (*n*=23) Statistical difference was determined via two-way ANOVA and reached significance with *P*< 0.05 (*) Significance is indicated for formerly obese against lean mice. (**b**) Lean mass and fat mass was determined in a subgroup of lean, obese and formerly obese mice (*n*=10–11), after 27 weeks of feeding and 35 weeks of age. (**c**) Intraperitoneal glucose tolerance (GTT) test (glucose 2 g per kg body weight) in a subgroup of formerly obese, lean and obese mice (*n*=11–12) at 35 weeks of age. (**d**) Area under the curve (AUC) of performed GTT (*n*=11–12). Data are presented as mean±standard error of the mean (s.e.m.).

To investigate molecular alterations caused by a history of obesity, we studied age-matched obese, formerly obese and lean mice. To this end, 8-week-old, male C57BL/6J mice were fed a high-fat high-sucrose diet (HFD) for 20 weeks. After 20 weeks of feeding, HFD-fed mice gained significantly more weight than mice fed a control diet (32.75 g±0.74 g vs 47.84 g±1.57 g, *P*<0.0001). The *ad libitum* switch from HFD to low-fat diet (LFD) induced a significant weight reduction, already within 1 week after the switch (47.62 g ±1.12 g vs 43.33 g±1.19 g, *P*<0.05). Weight loss continued in these formerly obese mice during the following 5 weeks, while mice on HFD continuously gained weight (Figure 1a). After 27 weeks on the experimental diet, the body weight of formerly obese mice was not statistically different from lean control mice (37.82 g ±1.26 g vs 34.84 g±0.89 g) (Figure 1a).

Weight loss in formerly obese mice was accompanied by reduced caloric intake (Supplementary Figure 1A), but no differences in browning of scWAT (Supplementary Figure 1B) compared to obese mice, or expression of UCP-1, Pgc1α (Supplementary Figure 1C) and oxygen consumption in BAT (Supplementary Figure 1D).

Consistent with the normalization of body mass, total lean and fat mass were reduced in formerly obese mice, and were not different to those of lean mice (Figure 1b).

After 27 weeks on the experimental diets, HFD-fed obese mice had impaired glucose tolerance, which was not observed in formerly obese and lean mice (Figures 1c and d). Furthermore, the switch to LFD induced a significant reversion of circulating levels of triglycerides, cholesterol, non-esterified free fatty acids, leptin, adiponectin and insulin (Supplementary Table 1).

### Depot-specific adipose tissue alterations after weight loss

**Figure 2:**
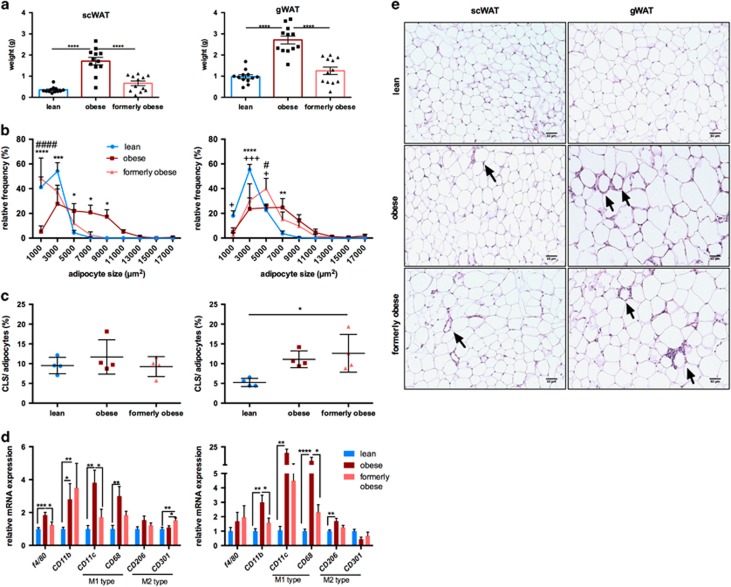
Depot-specific adipose tissue heterogeneity after a history of obesity. (**a**) Subcutaneous (scWAT) and perigonadal (gWAT) tissue wet weight (g) of lean, obese and formerly obese mice after 27 weeks of experimental diet (*n*=12). (**b**) Frequency distribution of adipocyte cell sizes (μm^2^) from scWAT and gWAT of lean, obese and formerly obese mice (*n*=4–5). Significant differences are indicated with+(formerly obese vs lean mice), # (obese vs formerly obese) and *=*P*<0.05 (obese vs lean) and were determined via two-way ANOVA followed by Tukey’s multiple comparison test. (**c**) % of counted crown-like structures (CLS) per number of counted adipocytes of scWAT and gWAT (*n*=4–5). (**d**) qPCR analysis of macrophage markers in scWAT (left panel) and gWAT (right panel). Expression levels are normalized to housekeeping gene TBP and shown as fold-change compared to the lean group (*n*=7–11). (**e**) Representative sections from H&E-stained scWAT (left panel) and gWAT (right panel) of lean, obese and formerly obese mice (× 200 magnification, scale bar=50 μm). Arrows indicate CLS.

Previous studies reported conflicting effects of weight loss on adipose tissue depots.^29, 36^ As expected, weight loss reduced the mass of both subcutaneous (scWAT) and perigonadal (gWAT) white adipose tissue to the level of lean mice (Figure 2a). Adipocyte size in both depots was increased upon prolonged HFD feeding compared to lean control mice. However, weight loss in formerly obese mice resulted in reduced adipocyte size in subcutaneous but not visceral depots after 7 weeks of diet change (Figure 2b). Quantification of crown-like structures, indicative of macrophage infiltration and inflammation,^37^ in sections of scWAT and gWAT revealed a perigonadal-specific increase in crown-like structure in obese mice, which was not reduced in tissue sections from formerly obese mice (*P*<0.05) (Figures 2c and d, e). Analysis of different markers for macrophages (Figure 2d) and inflammation (Supplementary Figure 2) showed an increase in *F4/80*, *Cd11b*, *Cd11c*, *Cd68* as well as proinflammatory cytokines *Il6* and *Tnf* in both adipose tissue depots of obese mice, which was largely reversed in formerly obese mice. Expression of *Cd11b* and the M2 macrophage marker *Cd301* were increased in formerly obese compared to lean mice in scWAT. Based on qPCR analysis, leptin mRNA levels were augmented in scWAT, but not in gWAT of obese mice and reduced in formerly obese mice. In contrast, adiponectin mRNA levels were reduced by obesity in gWAT, but not scWAT and not completely reversed by the diet switch, when comparing formerly obese vs lean mice (Supplementary Figure 2).

**Figure 3:**
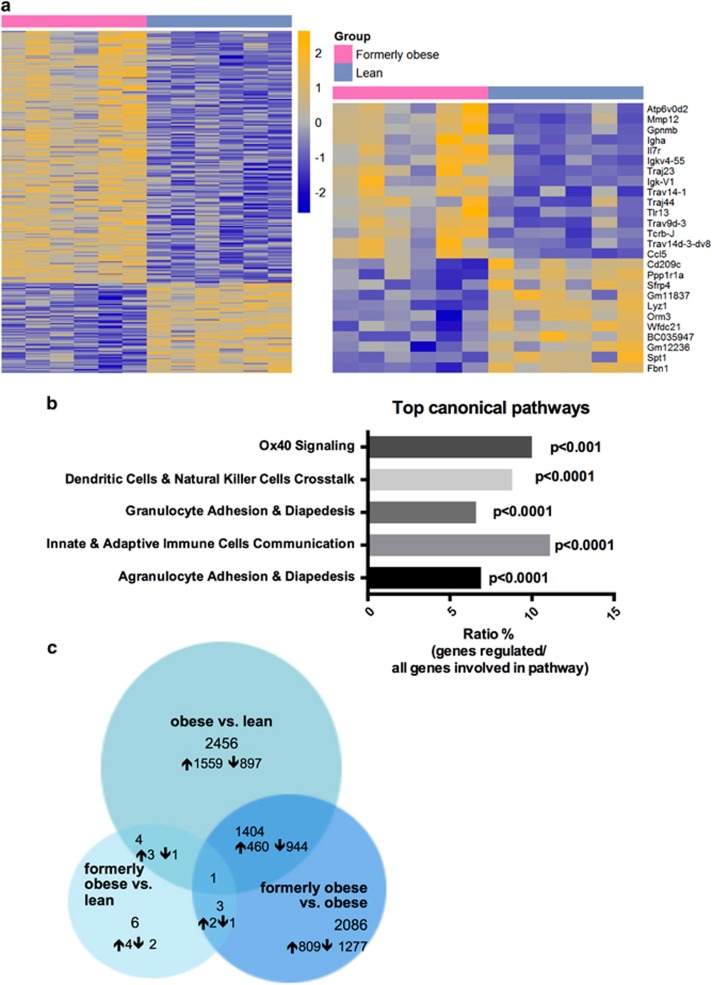
A history of diet-induced obesity covers a proinflammatory adipogenic transcription profile. (**a**) Heat map of 309 significantly (*P*<0.01) regulated genes in gWAT of formerly obese vs lean mice (*n*=6). Top up- and downregulated genes are presented separately. A detailed list of the 309 differentially expressed genes can be found in Supplementary Table 2. Statistical analysis was performed with the limma *t*-test and FC>1.3 ×, *P*<0.01 was considered as statistical significant. (**b**) Top five statistically significant (*P*<0.001) enriched canonical pathways associated with the 309 differentially expressed genes of formerly obese vs lean mice. A complete list of significantly enriched canonical pathways is attached in Supplementary Table 3. (**c**) Venn-diagram of (overlapping) significantly differentially expressed genes (FC>1.3, FDR<10%) in three pairwise comparisons ‘formerly obese vs lean’ (left panel), ‘obese vs lean’ (upper panel) and ‘formerly obese vs obese’ (right panel). A detailed list of the differentially expressed genes regarding each comparison can be found in Supplementary Tables 2, 4 and 5.

To further investigate the molecular alterations in the gWAT, we performed transcriptional profiling of the perigonadal adipose tissue of formerly obese, obese and lean mice, at the age of 35 weeks. In direct comparison between formerly obese and lean mice, we identified 309 differentially expressed genes (FC>1.3, *P*<0.01, Av>16), of which 234 were up-and 75 were downregulated in gWAT of formerly obese mice (Figure 3a and Supplementary Table 2). Among the top-regulated canonical pathways, we identified immunity- and inflammation-associated pathways such as ‘granulocyte adhesion and diapedesis’ pathways to be enriched (*P*<0.0001) (Figure 3b and Supplementary Table 3). A more stringent analysis of formerly obese vs lean mice (FC>1.3 ×, FDR<10%) narrowed down the number of regulated genes to six, which were associated with immune function (*Vcam1, Lyz1, H2-q5*), cellularity (*Fbn1*) and body weight regulation (*Nmb*) or have not been described before (*Gm26523*) (Figure 3c and Supplementary Table 2). Four of these identified candidate genes were also significantly regulated in the same direction when comparing obese and lean mice (*Gm26523*, *H2-q5*, *Vcam1* and *Lyz1*) (Figure 3c and Supplementary Tables 2 and 4).

Thus, weight loss and normalization of total fat mass only partially and depot-specifically restored adipocyte hypertrophy and obesity-associated inflammation. The persisting perigonadal hypertrophy was further paralleled by a differential regulation of genes, which were associated to immune function, cellularity and body weight regulation.

### *Ad libitum* weight loss partly reverses obesity-associated hepatic steatosis

**Figure 4:**
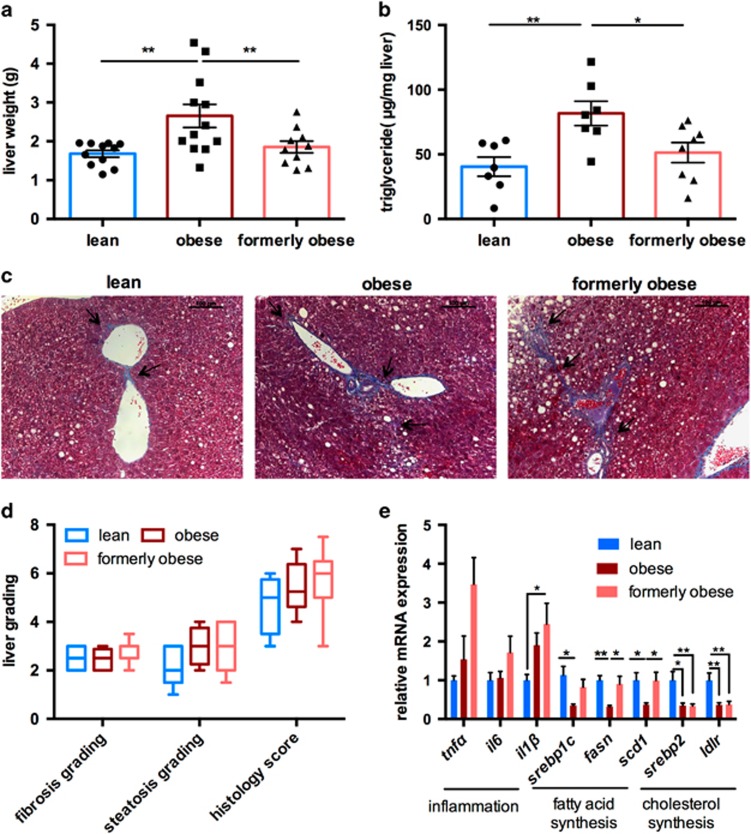
*Ad libitum* switch to low-fat diet partly reverses hepatic steatosis. (**a**) Liver weights of lean, obese and formerly obese mice, after 27 weeks of feeding the experimental diets (*n*=10–12). (**b**) Triglyceride contents (μg per mg of liver tissue) from livers of lean, obese and formerly obese mice (*n*=7–8). (**c**) Representative Masson’s trichrome-stained liver sections of a lean, obese and formerly obese mouse (× 200 magnification, scale 100 μm). Arrows indicate fibrotic lesions around the portal area and portal-to-portal bridging. (**d**) Histological scoring of H&E- and Masson’s Trichrome-stained liver sections (*n*=5–8). Data are given as boxplots indicating mean, minimum and maximum. (**e**) QPcr analysis of hepatic genes. Expression levels are normalized to housekeeping gene HPRT and shown as fold-change compared to the lean group. Data are presented as mean±standard error of the mean (s.e.m.) and analyzed using one-way ANOVA followed by Tukey’s multiple comparison test (*P<0.05, **P<0.01).

Prolonged HFD feeding and adipocyte dysfunction results in hepatic steatosis, as the first step towards NAFLD. To assess the grade of fatty liver disease, we first determined liver weight and hepatic triglyceride content in the livers of lean, obese and formerly obese mice, at the age of 35 weeks (Figures 4a and b). HFD feeding significantly increased liver weight and triglycerides content when compared to lean mice, which was completely reversed in formerly obese mice (Figures 4a and b). Hepatic steatosis is often accompanied by inflammation and fibrotic lesions, eventually progressing towards NAFLD. We determined the grade of fibrosis and the overall histology score using Masson’s trichrome-stained liver sections, according to standard guidelines,^31^ and detected slight portal to portal- and pericellular fibrosis in formerly obese mice, which was more comparable to those of obese, than lean mice (Figures 4c and d).

Real-time PCR confirmed increased expression of the proinflammatory cytokine *Il1ß* in the livers of formerly obese vs lean mice (*P*<0.05) (Figure 4e). In contrast, expression of genes involved in fatty acid uptake and *de novo* lipogenesis were only reduced in obese mice, whereas *Srebp2* and low-density lipoprotein receptor (*Ldlr*) were persistently downregulated in the livers of obese and formerly obese mice, when compared to lean mice (Figure 4e).

**Figure 5:**
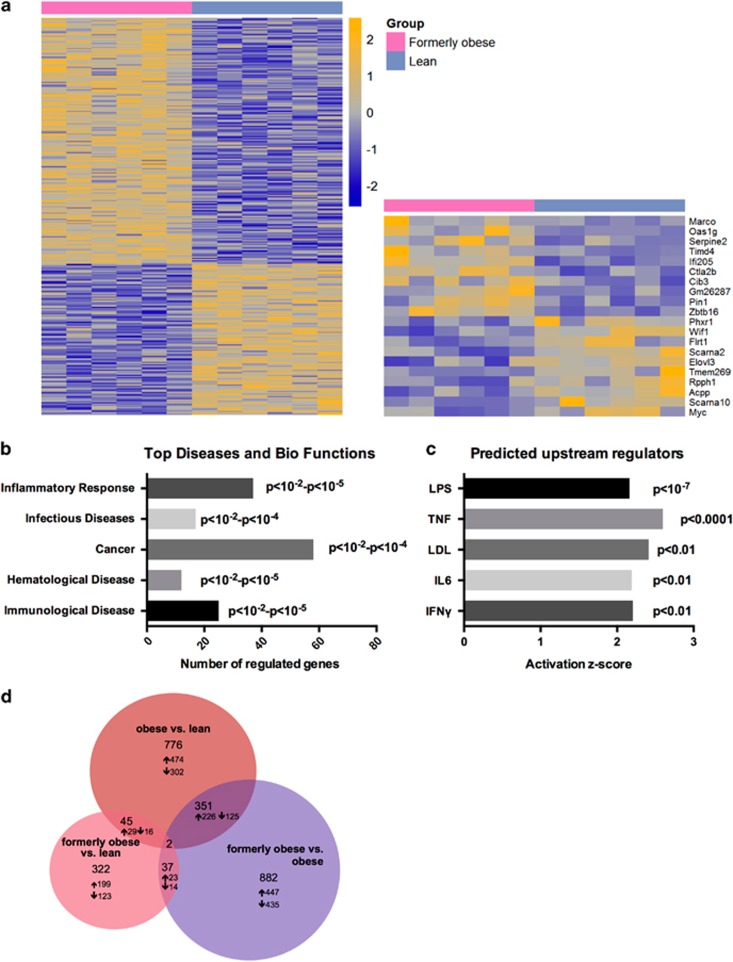
A history of diet-induced obesity covers a proinflammatory hepatic transcription profile. (**a**) Heat map of 322 significantly (*P*<0.01) regulated hepatic genes in the comparison of formerly obese and lean mice (*n*=6). Top up- and downregulated genes are presented separately. A detailed list of the 322 differentially expressed genes is attached in Supplementary Table 6. (**b**) Top five statistically significant (by *P*-value; *P*<0.01) activated ‘Diseases and Bio Functions’ associated with the 322 regulated genes. A detailed list of annotated terms can be found in Supplementary Table 7. (**c**) Significantly activated predicted upstream regulators of the 322 regulated hepatic genes. A list of all predicted activated upstream regulators of the 322 differentially expressed genes can be found in Supplementary Table 8. (**d**) Venn-diagram of (overlapping) differentially expressed genes (FC>1.2 *P*<0.01) in three pairwise comparisons ‘formerly obese vs lean’ (left panel), ‘obese vs lean’ (upper panel) and ‘formerly obese vs obese’ (right panel). A detailed list of the differentially expressed genes regarding each comparison can be found in Supplementary Tables 6, 9–13.

Gene expression profiling in livers of lean and formerly obese mice further identified 322 differentially regulated genes, of which 199 were significantly up- and 123 downregulated (FC>1.2, *P*< 0.01) (Figure 5a and Supplementary Table 6). Moreover, Ingenuity Pathway Analysis software identified ‘inflammatory response’ to be the top-associated ‘disease and bio-function’ pathway (*P*<0.01—*P*<10^−5^) (Figure 5b and Supplementary Table 7). Furthermore, we identified LPS, TNF, LDL, IL6 and IFNγ as predicted ‘activated upstream regulators’ of the 322 differentially regulated hepatic genes (Figure 5c and Supplementary Table 8). We further identified 45 genes, which were significantly regulated under obese and formerly obese conditions compared to lean mice (Figure 5d and Supplementary Table 9). These include proinflammatory cytokine *CCL5* and *Il2r*.

Thus, histological scoring and qPCR analysis revealed increased inflammation and fibrosis and a downregulation of genes involved in the cholesterol synthesis pathway, associated with a history of obesity. Moreover, 322 hepatic genes were differentially regulated by a history of obesity and enriched for inflammatory genes.

### Weight loss does not induce hyperphagia within 48 h of HFD re-feeding

**Figure 6:**
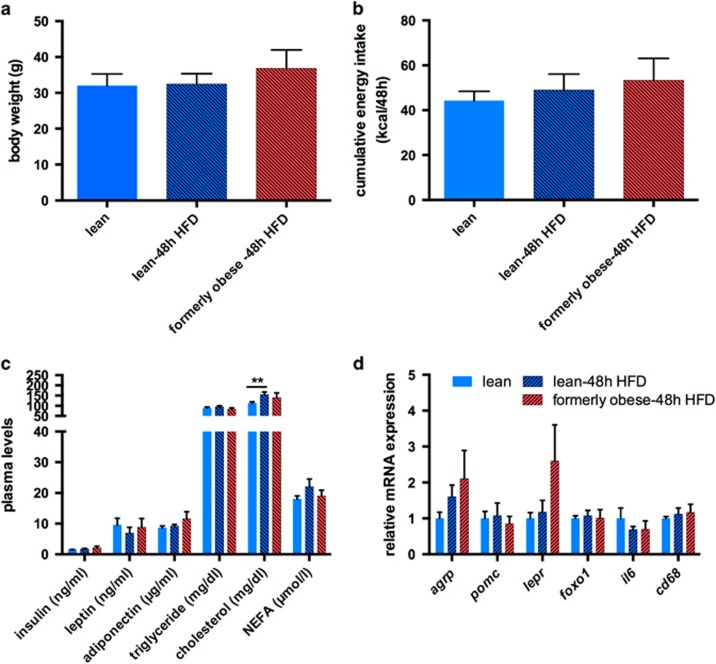
A history of diet-induced obesity does not facilitate hyperphagia within 48 h of re-feeding. (**a**) Body weight of lean and formerly obese mice, (re-) fed with HFD for 48 h (*n*=12). (**b**) Cumulative energy intake from lean and formerly obese mice, (re-) fed with HFD for 48 h (*n*=6) calculated as kcal/48 h/2 mice. (**c**) Plasma metabolites of lean and formerly obese mice (re-) fed with HFD for 48 h (*n*=8). (**d**) mRNA expression of hypothalamic orexigenic and inflammatory genes lean and formerly obese mice (re-) fed with HFD for 48 h and obese mice (*n*=6). Data are given as mean±standard error of the mean (s.e.m.). Statistical significance was indicated with *=*P*<0.05.

Well-known characteristics of weight loss upon caloric restriction and re-exposure to HFD are hyperphagia and excessive weight gain.^27, 36^ Thus, cohorts of lean and formerly obese mice (*n*=12) were (re-) introduced to hypercaloric HFD feeding for 48 h, at 35 weeks of age. We did not observe a significant difference in cumulative energy intake or weight gain between formerly obese and lean mice during the 48 h of re-feeding (Figures 6a and b). Moreover, re-feeding did not induce any significant alteration in circulating levels of insulin, leptin, adiponectin, triglycerides, cholesterol or free fatty acids between formerly obese and lean mice (Figure 6c). In contrast, 48 h of HFD-feeding increased plasma cholesterol in lean mice to such an extent that we could not detect any differences to obese mice any more (lean-48 h 154.16±13.34 mg dl^−1^ vs obese 184.74±17.59 mg dl^−1^) (Figure 6c and Supplementary Table 1). We did not detect differences in transcription of *Srebp2* or *Ldlr* in the livers of lean and formerly obese mice, upon 48 h re-feeding of HFD (Supplementary Figure 3). In line with the absence of hyperphagia in formerly obese mice we did not detect any significant differences in the hypothalamic expression of the orexigenic neuronal marker Agouti-related peptide *Agrp* and anorexigenic marker Proopiomelanocortin *Pomc,* compared to lean mice (Figure 6d). Moreover, expression of the leptin receptor (*Lepr*) and *Foxo1*, both downstream targets of leptin, were not statistically different between formerly obese and lean control mice (Figure 6d). In addition to the effects on food intake, short-term HFD feeding causes hypothalamic inflammation.^38^ However, neither expression of *Il6* nor *Cd68* was significantly altered between lean and formerly obese mice (Figure 6d). Thus, former obesity did not increase hyperphagia, body weight rebound or markers of hypothalamic inflammation within 48 h of HFD feeding.

## Discussion

Changes in diet and exercise are the preferred method of weight loss for most people.^12, 18, 39, 40^ Yet, sustained weight loss following dietary interventions is rarely achieved and most subjects regain an even higher body weight than pre-dieting within 1 year.^19, 22^ More importantly, however, the beneficial metabolic effects of weight loss are rapidly diminished by regaining even only a fraction of the pre-dieting weight. This suggests a metabolic memory of past obesity that persists through weight loss and predisposes to the development of the metabolic syndrome upon weight regain.

Using a model of voluntary caloric restriction in diet-induced obese mice, we aimed to investigate the molecular alterations inherited from a history of obesity. In line with previous studies we show that weight loss, induced by an *ad libitum* switch to a LFD, normalizes many of the diet-induced obese phenotypes of male C57BL/6J mice, including a reduction of total body- and fat mass, glucose tolerance and circulating metabolic parameters, within 7 weeks after the change of diet. However, similar to a recent report,^29^ we find persistent perigonadal adipose tissue inflammation in formerly obese mice. Extending previous reports, we show that adipocyte hypertrophy persisted in perigonadal adipose tissue and provide a detailed transcriptional analysis of genes regulated in formerly obese vs lean mice. Moreover, we find that former obesity leaves a specific inflammatory imprint in the liver, associated with altered gene expression and increased fibrosis, whereas in contrast to other models of weight loss^25, 27^ we did not observe hyperphagia or signs of hypothalamic inflammation upon re-exposure to HFD for 48 h.

It is well established that visceral and subcutaneous adipose tissue exhibit a depot-specific response to weight gain,^8^ which is associated with individual metabolic risks.^4^ In principle, adipocyte size positively correlates with fat mass.^41^ However, cell size of subcutaneous adipocytes inversely correlates with insulin sensitivity and the rate of weight loss in formerly obese mice.^42, 43^ Increases in visceral adipocyte size is associated with increased local and systemic inflammation and insulin resistance.^44^

Leptin, preferentially expressed in subcutaneous adipose tissue,^45^ is central to the regulation of body weight and leptin resistance significantly contributes to excessive weight gain. In line with previous studies by Enriori *et al.*,^46^ the restoration of body and fat mass reversed obesity-related hyperleptinemia in formerly obese mice. However, both caloric restriction and bariatric surgery do not improve visceral adipose tissue inflammation within 1 year post-intervention in mice and obese patients.^29, 36^ Our data confirm and extend these previous observations as we find persistent inflammation, as evidenced by our transcriptome analysis as well as an increased number of crown-like structures in gWAT. Moreover, albeit overall perigonadal adipose tissue mass is reduced to the level of lean mice, individual adipocyte size remained enlarged and comparable to those of obese mice. This indicates that, as supported by our network analysis, weight loss results in the loss of perigonadal adipocytes, requiring extensive tissue remodeling. In contrast, reduction in subcutaneous adipose tissue mass seems to be largely driven by an overall reduction of stored triglycerides within individual adipocytes. This is also accompanied by an increased expression of the lipolysis-associated M2 macrophage marker *Cd301*.^47, 48^ To this end, it will be interesting to see if this loss of adipocytes is driven by adipocyte intrinsic mechanisms or through inflammatory cells such as macrophages and to what extent the genes identified to be regulated in perigonadal fat of formerly obese mice (*Vcam1*, *Nmb*, *lyz1*, *Gm26523*, *Fbn1* and *H2-q5*) contribute to this process.

As discussed above, previous studies described some persistent alterations in adipose tissue and impaired systemic insulin sensitivity upon weight loss.^13, 29, 36^ Most of these studies concluded that adipose tissue is the major driver of this systemic insulin resistance with little contribution of the liver.^29^ These conclusions were corroborated by studies in mice and men demonstrating a beneficial effect of weight loss on the outcome of NAFLD.^49, 50, 51^ However, these assessments were largely based on the reduction of liver triglycerides upon weight loss, suggesting a restoration of liver function. In addition to steatosis, prolonged HFD feeding also induces other hallmarks of NAFLD, such as inflammation, fibrosis or hepatocellular ballooning.^52^ Similar to previous studies,^53^ we find increased liver weights, triglyceride contents, accumulation of macrovesicular fat droplets, increased inflammatory marker expression and portal-to portal as well as pericellular, so-called ‘chicken-wire fibrosis’ in obese mice.

As expected, weight loss reduced liver triglyceride levels to that of lean control animals. However, the histology score remained comparable to obese mice. The differences between total liver triglyceride content and steatosis grading, from the histological slides, reflect differences in the regional lipid distribution of formerly obese mice, further underscoring the importance to use both methods in parallel. Enhanced expression of the inflammatory cytokine *Il1β* was further confirmed by microarray-based transcriptomics from livers of formerly obese and lean mice, where the top-associated disease was ‘inflammatory response’ and predicted ‘activated upstream regulators’ were TNF and IL6. Our study was terminated 7 weeks following the diet switch; thus we cannot exclude that some or all of these inflammatory events are lost over time, as fibrosis and inflammation can also be indicators of tissue regeneration.^54^ Nevertheless, our data show that proinflammatory signals together with increased histological alterations are maintained even after restoration of a lean body weight. To this end, it is very likely that the liver is central to the detrimental consequences of weight regain following moderate weight loss. Thus it is tempting to speculate that maintaining a lean body weight following weight loss is critically important to prevent any of the persisting molecular alterations in adipose tissue and the liver to negatively impact on metabolism. Importantly, however, the onset and development of obesity and the metabolic syndrome is sex dependent.^55, 56^ Despite a higher prevalence of obesity, the prevalence of NAFLD is lower in women than in men.^55^ Moreover, female mice are less susceptible to diet-induced obesity and fatty liver disease.^57^ Thus, it will be interesting to study the consequences of *ad libitum* weight loss in models of female obesity, hepatosteatosis and insulin resistance in the future. Nevertheless, most humans regain most or even more than their pre-dieting weight within a relatively short time after dieting.^19, 20, 21^ We and others have previously shown that caloric restriction induces acute and persistent hyperphagia and body weight rebound of formerly obese mice upon *ad libitum* re-feeding of both, low- and high-fat diets.^25, 27, 36^ Interestingly, however, the current model of *ad libitum* switch to an LFD did not accelerate food intake nor body weight gain upon re-exposure to HFD for 48 h. This could in part be due to differences in experimental set-ups, as in this study mice were only fasted for 3 h before re-exposure to HFD and not 24 h, like previously reported.^27^ Moreover, we did not detect significantly altered expression of *Agrp*, *Pomc*, *Foxo1* and *Leptinr* nor increased hypothalamic inflammation, when comparing formerly obese to lean mice exposed to HFD.

Hence, in contrast to severe caloric restriction, *ad libitum* switch to a low-caloric diet does not induce strong orexigenic hypothalamic alterations, which facilitate body mass rebound. Thus, the degree of hyperphagia seems to be dominated by the availability of food rather than the calories consumed.

In conclusion, weight loss due to a simple switch from an HFD to an LFD reverses many metabolic phenotypes in mice within 2 months. Moreover, this weight loss model is not associated with immediate hyperphagia upon re-exposure to HFD, indicating that consuming food choices with a reduced caloric value *ad libitum* instead of strict caloric restriction may present an attractive strategy to successfully reduce and maintain body weight. However, perigonadal fat, as one of the most prominent visceral adipose tissue depots in mice, and the liver maintain a molecular memory of a history of obesity. Both of these tissues are critically important for the development of insulin resistance and glucose intolerance. Thus, future weight loss studies should include monitoring and treatment of these inflammatory processes after the diet intervention to prevent a deterioration of metabolic health upon moderate weight regain.
