# Supplementary material for: A history of obesity leaves an inflammatory fingerprint in liver and adipose tissue
Source: Int J Obes (Lond). 2017 Oct 24;42(3):507–17. doi: 10.1038/ijo.2017.224 (PMC5880583; doi:10.1038/ijo.2017.224)
Supplement: Supplementary Information [file ijo2017224x1.docx]

**Text summary of Supplementary Information:**

**Supplemental Figure 1**: Caloric intake and qPCR data from scWAT and BAT of lean. Obese and formerly obese mice.

**Supplemental Figure 2:** relative mRNA expression levels of cytokines and adipokines measured in scWAT and gWAT of lean, obese and formerly obese mice.

**Supplemental Figure 3**:relative mRNA expression levels of hepatic genes of lean and 48h-HFD refed animals

**Supplemental Table 1:** results of blood measurements of lean, obese and formerly obese mice.

**Supplemental Tables 2-13**: lists of differentially expressed genes and associated enriched pathways in gWAT and liver.

**Supplemental Table 14** : primer sequences used for qPCR analysis.
