# Supplementary figures and images for: A history of obesity leaves an inflammatory fingerprint in liver and adipose tissue
Source: Int J Obes (Lond). 2017 Oct 24;42(3):507–17. doi: 10.1038/ijo.2017.224 (PMC5880583; doi:10.1038/ijo.2017.224)

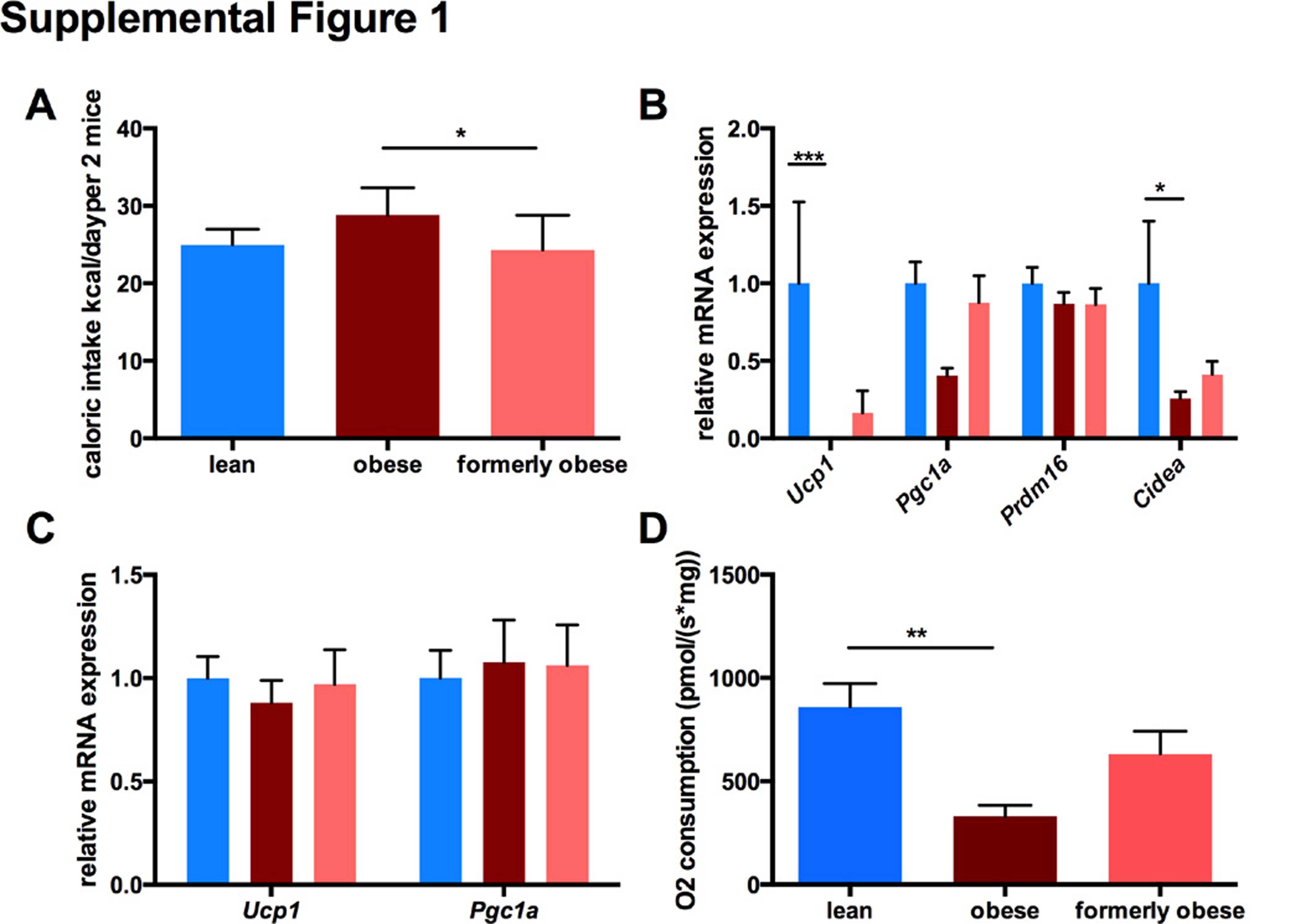

Supplement: Supplementary Figure 1 [file ijo2017224x2.tif]

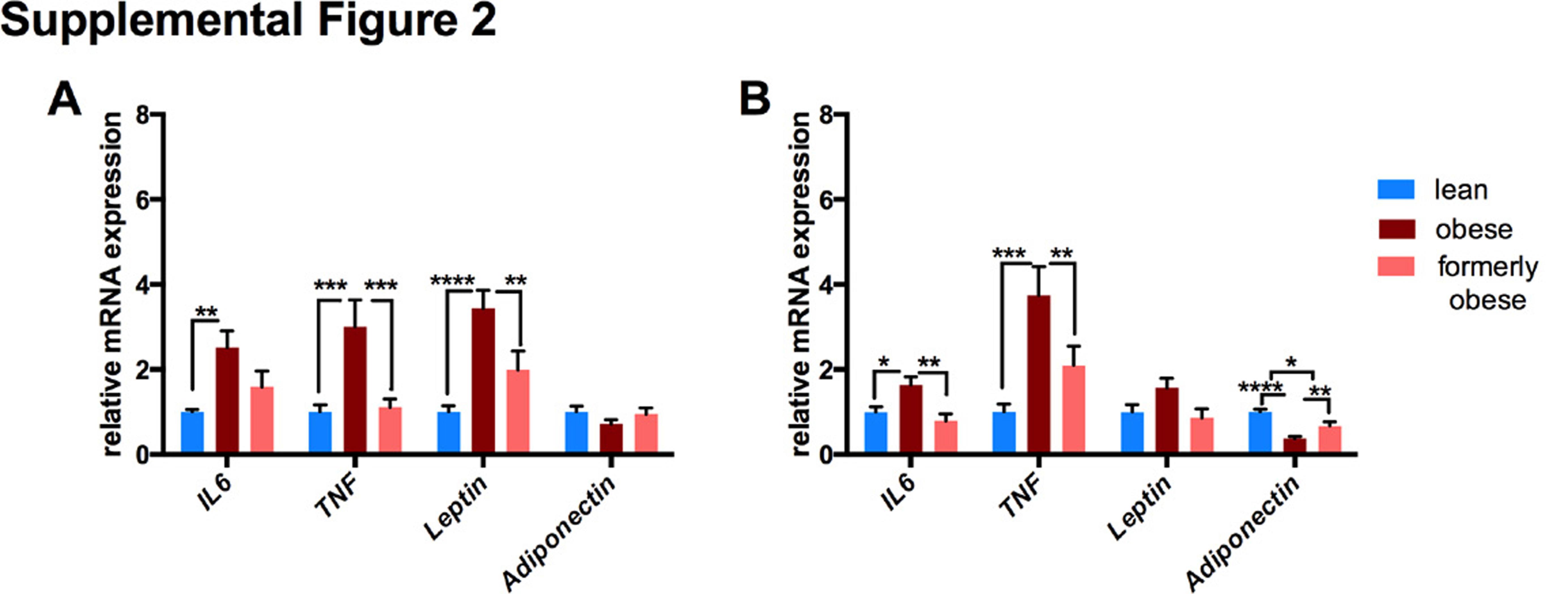

Supplement: Supplementary Figure 2 [file ijo2017224x3.tif]

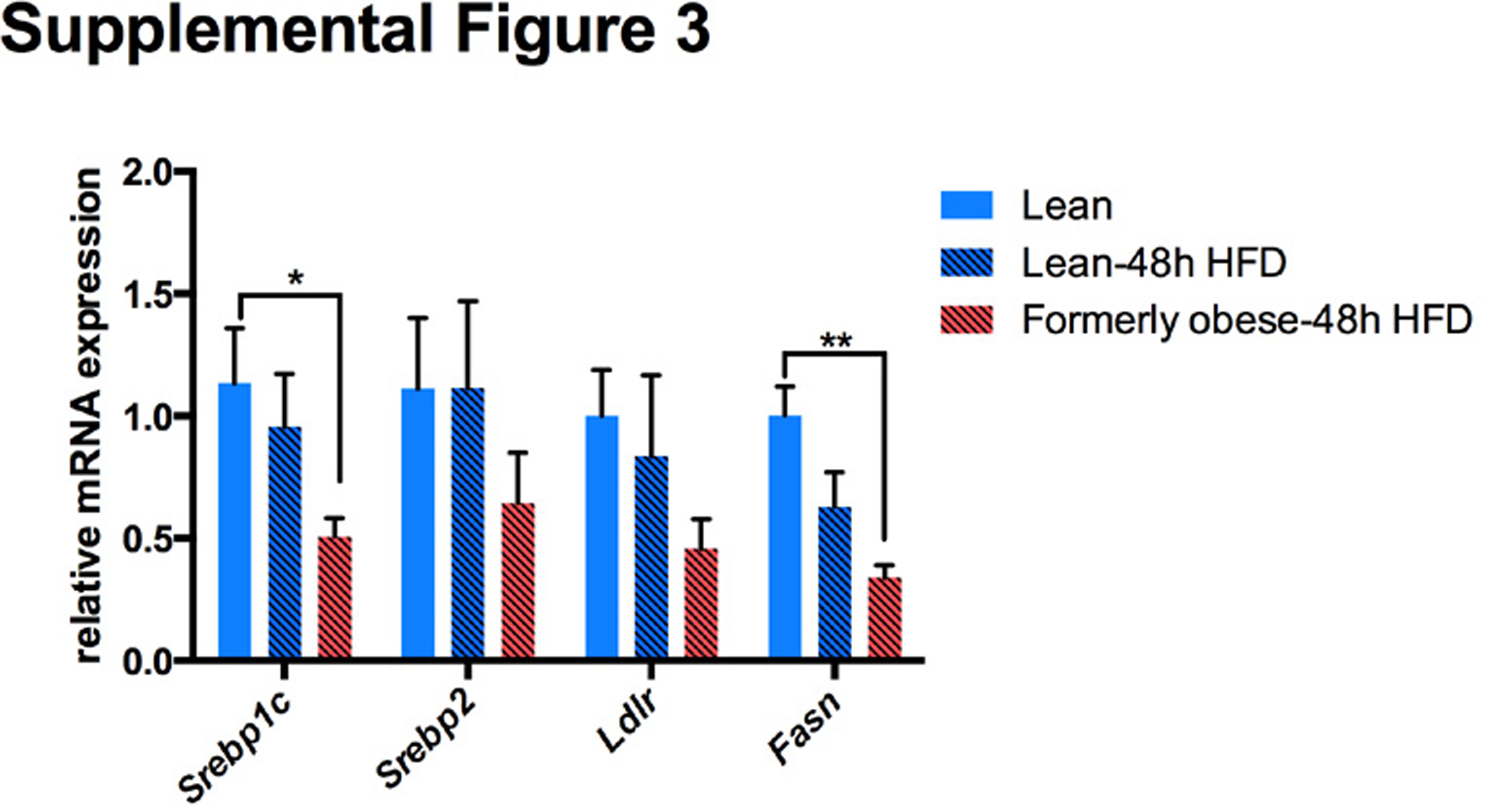

Supplement: Supplementary Figure 3 [file ijo2017224x4.tif]
