# Supplementary material for: A history of obesity leaves an inflammatory fingerprint in liver and adipose tissue
Source: Int J Obes (Lond). 2017 Oct 24;42(3):507–17. doi: 10.1038/ijo.2017.224 (PMC5880583; doi:10.1038/ijo.2017.224)
Supplement: Supplementary Table 1 [file ijo2017224x5.docx]

Supplemental Table 1: Plasma metabolites after 27 weeks of feeding experimental diets.

|  | **lean** | **obese** | **formerly obese** |
| --- | --- | --- | --- |
| **Triglyceride (mg/dl)** | 81.43±5.84^++^ | 121.0±11.41^*^ | 62.0 ± 8.06^+++^ |
| **Cholesterol (mg/dl)** | 112.3 ± 7.06^++^ | 184.7 ± 17.59^**^ | 103.5 ±1 6.74^++^ |
| **NEFA (µmol/l)** | 17.94 ± 1.07 | 22.13 ± 1.04 | 14.61 ± 2.64^+^ |
| **Insulin (ng/ml)** | 1.41 ± 0.19^++++^ | 8.01 ± 1.04^****^ | 1.75 ± 0.34^++++^ |
| **Leptin (ng/ml)** | 9.52 ± 2.21^++++^ | 38.52 ±4.97^****^ | 10.33 ± 2.97^++++^ |
| **Adiponectin (µg/ml)** | 8.59 ± 0.67 | 7.54 ± 0.50 | 10.51 ± 0.83^+^ |

*p<0.05, **p<0.01, ***p<0.001, ****p<0.0001 compared to lean mice; ^+^p<0.05, ^++^p<0.01, ^+++^p<0.001, ^++++^p<0.0001 compared to obese mice. Data were analyzed using One-way ANOVA followed by Tukey’s multiple comparison test. N=7-8 per plasma metabolites analyses. Data are presented as mean ± standard error of the mean (SEM).
