# Supplementary material for: A history of obesity leaves an inflammatory fingerprint in liver and adipose tissue
Source: Int J Obes (Lond). 2017 Oct 24;42(3):507–17. doi: 10.1038/ijo.2017.224 (PMC5880583; doi:10.1038/ijo.2017.224)
Supplement: Supplementary Table 14 [file ijo2017224x10.docx]

**Supplemental Table 14**

**Primer List**

| **Genename** | **Forward (5’<3’)** | **Reverse (3’<5’)** |
| --- | --- | --- |
| *Agrp* | GGCCTCAAGAAGACAACTGC | GCAAAAGGCATTGAAGAAGC |
| *Pomc* | CATTAGGCTTGGAGCAGGTC | TCTTGATGATGGCGTTCTTG |
| *Lepr* | CGTGGTGAAGCATCGTACTG | GGGCCATGAGAAGGTAAGGT |
| *Foxo1* | CGCCTCCTACTACTGAGC | TGTCTGTACTTAGGCGCACA |
| *Il6* | TAGTCCTTCCTACCCCAATTTCC | TTGGTCCTTAGCCACTCCTTC |
| *Cd68* | TGTCTGATCTTGCTAGGACCG | GAGAGTAACGGCCTTTTTGTGA |
| *F4/80* | CATAAGCTGGGCAAGTGGTA | GGATGTACAGATGGGGGATG |
| *Cd11b* | TGACCTGGCTTTAGACCCTG | ACCTCTGAGCATCCATAGCC |
| *Cd11c* | CTGGATAGCCTTTCTTCTGCTG | GCACACTGTGTCCGAACTCA |
| *Cd206* | TGATTACGAGCAGTGGAAGC | GTTCACCGTAAGCCCAATTT |
| *Cd301* | CTCTGGAGAGCACAGTGGAG | ACTTCCGAGCCGTTGTTCT |
| *Ucp1* | GGCCTCTACGACTCAGTCCA | TAAGCCGGCTGAGATCTTGT |
| *Pgc1α* | AGCCGTGACCACTGACAACGAG | GCTGCATGGTTCTGAGTGCTAAG |
| *Prdm16* | CCGCTGTGATGAGTGTGATG | GGACGATCATGTGTTGCTCC |
| *Cidea* | AATGGACACCGGGTAGTAAGT | CAGCCTGTATAGGTCGAAGGT |
| *Leptin* | GGGCTTCACCCCATTCTGA | TGGCTATCTGCAGCACATTTTG |
| *Adiponectin* | TGTTCCTCTTAATCCTGCCCA | CCAACCTGCACAAGTTCCCTT |
| *Srebp1c* | GGAGCCATGGATTGCACATT | GGCCCGGGAAGTCACTGT |
| *Fasn* | AGAGATCCCGAGACGCTTCT | GCTTGGTCCTTTGAAGTCGAAGA |
| *Il1β* | GCAACTGTTCCTGAACTCAACT | ATCTTTTGGGGTCCGTCAACT |
| *Scd1* | TCCAAGCGCAGTTCCGCCAC | TGGAGATCTCTTGGAGCATGTGG |
| *Srebp2* | CATTCTCCAGCAGTTCCGTG | GCCCTCTCACAGTGACAGAA |
| *Ldlr* | TCAGACGAACAAGGCTGTCC | CCATCTAGGCAATCTCGGTCTC |
| *Tnf* | CAGGCGGTGCCTATGTCTC | CGATCACCCCGAAGTTCAGTAG |
